# A Critical Interpersonal Distance Switches between Two Coordination Modes in Kendo Matches

**DOI:** 10.1371/journal.pone.0051877

**Published:** 2012-12-20

**Authors:** Motoki Okumura, Akifumi Kijima, Koji Kadota, Keiko Yokoyama, Hiroo Suzuki, Yuji Yamamoto

**Affiliations:** 1 Faculty of Education, Shizuoka University, Shizuoka, Japan; 2 Graduate School of Education, Shizuoka University, Shizuoka, Japan; 3 Graduate School of Education, University of Yamanashi, Yamanashi, Japan; 4 Department of Health and Sport Sciences, Graduate School of Medicine, Osaka University, Osaka, Japan; 5 Division of Applied Physics, Faculty of Engineering, Hokkaido University, Hokkaido, Japan; 6 Japan Society for the Promotion of Science, Tokyo, Japan; 7 Graduate School of Education and Human Development, Nagoya University, Nagoya, Japan; 8 Research Center of Health, Physical Fitness and Sports, Nagoya University, Nagoya, Japan; 9 Department of Psychology and Human Developmental Sciences, Nagoya University, Nagoya, Japan; McMaster University, Canada

## Abstract

In many competitive sports, players need to quickly and continuously execute movements that co-adapt to various movements executed by their opponents and physical objects. In a martial art such as kendo, players must be able to skillfully change interpersonal distance in order to win. However, very little information about the task and expertise properties of the maneuvers affecting interpersonal distance is available. This study investigated behavioral dynamics underlying opponent tasks by analyzing changes in interpersonal distance made by expert players in kendo matches. Analysis of preferred interpersonal distances indicated that players tended to step toward and away from their opponents based on two distances. The most preferred distance enabled the players to execute both striking and defensive movements immediately. The relative phase analysis of the velocities at which players executed steps toward and away revealed that players developed anti-phase synchronizations at near distances to maintain safe distances from their opponents. Alternatively, players shifted to in-phase synchronization to approach their opponents from far distances. This abrupt phase-transition phenomenon constitutes a characteristic bifurcation dynamics that regularly and instantaneously occurs between in- and anti-phase synchronizations at a critical interpersonal distance. These dynamics are profoundly affected by the task constraints of kendo and the physical constraints of the players. Thus, the current study identifies the clear behavioral dynamics that emerge in a sport setting.

## Introduction

Interpersonal distance constitutes a very important contributor not only to the psychological, physical, and social aspects of interactions [1] but also to the dynamics of interpersonal competition such as that in sports activities. Kendo, or Japanese fencing, is a martial art in which a player must strike an opponent using a bamboo sword (shinai) in a square court with 9.00–11.00-m sides. The player must simultaneously avoid a counterstrike from the opponent’s shinai. A point (ippon) in a kendo competition is achieved when an accurate strike is made on the opponent with the uppermost third, or the top 0.30–0.40 m of the total length, of the shinai (1.20 m). Typically, two to three points at most are scored during a 5-minute match, and the average time of a strike movement is 0.36 s (standard deviation (*SD*) 0.08 s) from an average interpersonal distance of 2.37 m (*SD*, 0.18) (see Text S1), and a split-second offensive or defensive maneuver may decide the outcome of the match. A player must carefully and constantly maintain and change interpersonal distances to balance the gain/loss of offensive and defensive maneuvers. Thus, according to offensive–defensive trade-offs, if the interpersonal distance decreases or increases, the potential offensive gain or defensive loss simultaneously increases or decreases, respectively, as a function of reaction and movement times. Some properties of human-interaction tasks might operate under common constraints in competitive tasks. Our aim was to describe the behavioral dynamics that are obscured in competitive one-on-one sporting situations such as kendo matches. To this end, a complete and simultaneous analysis of both players’ movements is necessary to elucidate the dynamics underlying the performance of tasks in such situations.

In an attempt to better understand human movement, Haken, Kelso, and Bunz [2] developed a model of rhythmic left and right index finger movements [3], [4] in relation to the relative phase difference in coupling strength between nonlinear coupled oscillators [5], [6]. A key feature of this nonlinear oscillator model is the frequency-induced transitions from the anti-phase to the in-phase synchronization of rhythmic finger movements. The relative phase between the two oscillators has bistability at in-phase and anti-phase synchronizations. This form of dynamic pattern formation involves the occurrence and eradication of stable behavioral patterns that can be described mathematically as attractors. It has been proposed that when a control parameter, such as movement frequency, reaches a certain critical value, the movement brings about a phase transition and shifts from bistability to monostability. This pattern switch attracts the movement into the in-phase synchronization, which is more stable than is the anti-phase synchronization [7]. Thus, these seminal studies clearly indicate that a critical change in the control parameter governs the regular change in the order parameter of the movement. The model developed by Haken, Kelso, and Bunz [2] has been applied to both intrapersonal and interpersonal coordination research.

In a study of interpersonal coordination, Schmidt et al. [8] asked paired participants to perform a simple rhythmic task in which they had visual information about each other’s movements and had to coordinate movement of their outer legs periodically and rhythmically. The participants’ movements were attracted into anti-phase synchronization and then into in-phase synchronization when their movements gradually increased in frequency and the control parameter reached a critical value. Such interpersonal coordination has been recently termed ‘joint action’ and has been examined in various tasks [9]. For example, interpersonal coordination was confirmed in the same manner [10] in cooperative tasks between paired participants such as in oscillation of a hand-held pendulum [11], [12], swaying of a rocking chair [13], cooperative pointing [14], and various social exchanges [15]–[18]. It has been proposed that interpersonal coordination occurs as a result of shared visual information within task constraints. Thus, interaction of the movements of two persons without physical contact has been demonstrated explicitly. Additionally, these previous studies showed that the nonlinear oscillator model has methodological advantages, including with regard to capturing changes in the temporal–spatial stream of continuous movements and exploring the dynamics underlying complex movements.

A number of researchers have suggested that use of this approach could provide insight into sports composed of competitive tasks and complex, continuous, and high-speed movements [17], [19]–[25]. However, in contrast to the attention devoted to cooperative tasks, little attention has been devoted to competitive tasks, and few researchers have analyzed movements in sporting games. According to previous studies on team games, the movements of playing dyads and teams are attracted to in-phase synchronization in the direction of the long axis on the court in basketball. Interestingly, the frequency of in- and anti-phase synchronizations in these studies differed depending on the individual pairings of players [26], [27]. Similarly, studies on rugby [28] and futsal [29], [30] found that relative positions of the ball and players, interpersonal distances between players, and relative velocities were all candidate control parameters that changed players’ movement patterns. However, because players in these games almost always not only cooperate with teammates but also compete against opponents at the same time and because the fields on which these games are played are often larger, the task constraints and the unfolding movements may differ essentially from those of martial arts or kendo.

Studies of one-on-one games such as tennis [31], [32] and squash [33] have found that players’ movements while taking turns hitting a ball frequently switched between in- and anti-phase synchronization in the direction of the short axis of the court. Thus, these studies have shown that players in competitive tasks vary their movements between in- and anti-phase synchronization in relation to ball/field position and/or interpersonal distance as candidate control parameters in the same manner observed in team games. However, in almost all ball-game situations, irrespective of whether they are played in teams or one-on-one, a team or a player continuously alternates between hitting and possessing a ball, and the direction of the players’ movements strongly depends on the course of the ball. Similarly, in most situations, the roles of offensive and defensive players are distinct and continue for periods of time. If these games are conceptualized in terms of an oscillation model, two players or teams could be considered to be strongly coupled nonlinear oscillators in certain situations. Thus, because the task constraints of the aforementioned games differ considerably from those of kendo, in which two players must change continuously and instantaneously between offensive and defensive roles, it cannot be concluded that kendo players and/or teams evenly compete and oppose each other in offensive–defensive trade-off situations.

The behavioral dynamics of competitive tasks, particularly those involving ‘opponent tasks’ such as kendo, remain unclear. Indeed, Dietrich et al. [34] modeled variation in the interpersonal distance between two players in kendo as a linear mass spring pendulum. In that study, it was assumed that the two players’ movements were self-independent and simulated a linear coupled harmonic oscillator that integrated players’ strategy intensity to step backward from and forward toward opponents. Although the player needs to co-adapt to the opponent’s movements, the two players cannot join together physically and can move freely around each other. Consequently, the two players can be categorized as weakly coupled nonlinear oscillators in opponent tasks.

In addition to lacking a clear understanding of the behavioral dynamics of opponent tasks, previous studies have also targeted only certain sub-phases of games and non-expert players. Consequently, some uncertainties remain as to whether previous studies assessing players’ movements truly reflected the properties of tasks and expertise [28], [31], [35]. It should be noted that some previous studies targeted sport games; however, few movements and only local parts of the game situations were analyzed [26], [27], [29], [30], [32]–[34]; thus, it was unclear whether the conclusions applied to the entire game situation and/or to the permanent dynamics of the competitive tasks. In other words, previous studies have confirmed that it is difficult to clearly identify the overall behavioral dynamics of sports, such as kendo, that involve various complex movements and situations. Further studies are needed to understand the common and unique dynamics of sports.

In response to the aforementioned issues, the current study examined the behavior of expert players during entire kendo matches using continuous changes in interpersonal distance. We assumed that interpersonal distance, as a candidate control parameter, would govern players’ movement patterns, especially those involving stepping toward and away from an opponent, which would act as an order parameter. Indeed, players must continue to skillfully maneuver interpersonal distance to balance the gains/losses in offense and defense under the constraints imposed by a close one-on-one situation, the very rapid pace at which movements (e.g., swinging and jumping) must be executed, the need to engage in cognitive (reaction and decision making) activities, and so on. Thus, a subtle change in interpersonal distance would be expected to lead to critical changes in the task constraints affecting, for example, the possibility of successful striking or defensive movements. Thus, maneuvers of this sort would be executed strategically rather than randomly. Like dancers, kendo players maintain a stable interpersonal distance from each other for periods of time. Like a mass-spring system, decreases in interpersonal distance occur suddenly, which renders the patterns of movements difficult to discern without experimental data. Our primary foci were on the relative phases of players’ step toward–away movements and on the behavioral dynamics of the opponent task. We hypothesized that hidden but clear dynamics reflected the task constraints on, and the meanings underlying, players’ movements, and that these movements would be characterized by continual abrupt changes between in- and anti-phase synchronizations depending on interpersonal distance. This study can contribute to understanding the dynamics not only of sport but also of other human social interactions.

## Materials and Methods

### Participants

Six male members of the University of Tsukuba kendo club participated in the experiment. This club has won the kendo championship in the annual team competition for all Japanese universities three times since the year 2000. All participants were healthy, regular players on the team; their average age was 20.67 (standard deviation (*SD*) of 0.75) years of age and they had an average of 14.17 (*SD* of 1.77) years of kendo experience. All participants provided written informed consent prior to the experiments. Procedures were approved by the Internal Review Board at the Research Center of Health, Fitness, and Sport at Nagoya University and conformed to the principles expressed in the Declaration of Helsinki.

### Tasks

#### Task 1: Matches

The six players were matched against four different opponents. If one player had been matched against five other players in a round-robin system, a total of 15 matches would have been played. However, because each player was not matched against one of the other players, a total of 12 matches were played. Following official kendo rules, each match lasted 5 minutes and was played on a square court with 11.00-m sides. Each match was judged by three referees. We observed 43.42 (*SD* of 8.40) instances of striking and 2.33 (*SD* of 1.25) points per a match.

#### Task 2: Possible striking distance

Measurements were taken after all matches to analyze the players’ ability and the physical constraints of a strike movement. These measurements required players to report the distances from which they could reasonably strike their opponents with a quick and brief movement. Players were able to strike their opponents from their own striking distances within about 0.50 s (see Introduction and Text S1). In other words, we considered the distance to indicate a kind of task constraint because players must move in accordance with their physical abilities. The measurement involved a player holding a shinai and stepping toward and away from an opponent who was standing and holding a shinai. The player would then determine and stop at his own striking distance. Two measurements were made in the middle of the court for each player in each match against four opponents, for a total of eight measurements.

### Measurement Procedures

#### Experimental devices

Players’ movement trajectories were recorded using an optical motion capture system with eight different cameras (100 Hz, OQUS300, Qualysis, Inc.) and a video camera placed around various positions on the court. Large reflective markers were attached to the back of the player’s head and right ankle and to the top of the shinai to detect movement (see below and Text S1).

#### Scene elimination

First, an experimenter eliminated unrelated scenes in which the match was stopped by the referees and those in which the reflective markers could not be seen in the images of the optical motion capture system because the players were outside the camera angles. As a result, the analyzed data averaged 4 min 19 s per a match with an *SD* of 17 s; thus, the matches actually lasted 5 minutes from start to finish.

## Dependent Measures

### Interpersonal Distance

The trajectory of the player’s head position was expressed as a time-dependent vector (*x*(*t*), *y*(*t*)) and (*u*(*t*), *v*(*t*)). These time-series vectors were calculated using software (Qualysis Track Manager, Qualysis, Inc.) and flattened using a fourth-order Butterworth filter with a cutoff frequency of 6 Hz. The time series for the Euclidean distance *D*(*t*) between two players was calculated using the following equation:
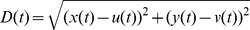
(1)


*D*(*t*) was calculated for the entire duration of each of the 12 matches.

#### Step toward–away velocity

**Figure 1:**
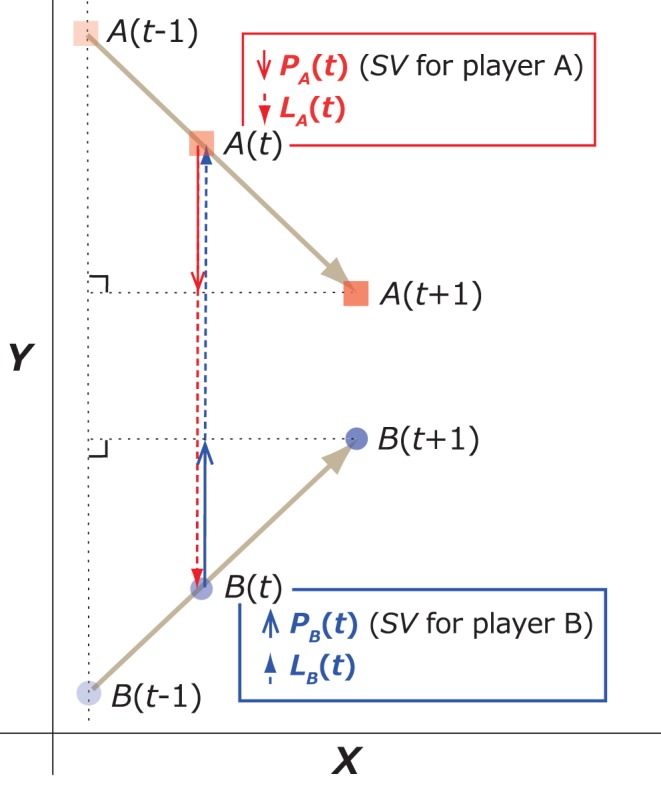
Schematic representation of step toward–away velocity (SV). In this example, player A moved from position *A*(*t*−1) to *A*(*t*+1) during *t* time from *t*−1 to *t*+1. *SV* for player A is depicted as *PA*(*t*) (red solid arrow) and was defined as the projection of vector *A*(*t*−1) → *A*(*t*+1) to vector *LA*(*t*) (red broken arrow). A negative sign of *SV* was assigned if the *SV* was directed toward the opponent, and a positive *SV* was assigned if the *SV* was directed away from the opponent. *SV* for player B was calculated using the same procedure (blue solid arrow). Note: Red and blue solid arrows denote the magnitude and direction of *SV*s for each player at *A*(*t*) and *B*(*t*), separately. In this example, the players moved primarily back and forth (along the *y*-axis) while facing each other. Although rare, lateral movements (along *x*-axis) were also observed and were included in the vector calculation.

Step toward–away velocity (*SV*) was calculated to estimate the movement of each player (hereafter, players A and B). To calculate these variables, the displacement vector from time *t*−1 to time *t*+1 (the sampling frequency was 100 Hz and the time lag in real time was 0.02 s) was defined as *A_t_*_−1*→t*+1_ = (*x*(*t* +1) − *x*(*t* − 1), *y*(*t* +1) − *y*(*t* − 1)) for player A and as *B_t_*_−1→*t*+1_ = (*u*(*t* +1) − *u*(*t* − 1), *v*(*t* +1) − *v*(*t* − 1)) for player B. The projection of vector *A_ t_*_−1→*t*+1_: (*P_A_*(*t*)) to a linked vector (for example, *L_A_*(*t*) = (*u*(*t*) − *x*(*t*), *v*(*t*) − *y*(*t*)) in Fig. 1) with the direction from the position of A to that of the position at B at time *t*, and that for the position of B (projection of vector *B_ t_*_−1→*t*+1_: *P_B_*(*t*); linked vector: *L_B_*(*t*) = (*x*(*t*)−*u*(*t*), *y*(*t*)−*v*(*t*))) were also obtained separately with equations (2) and (3) (see Fig. 1):
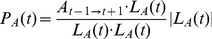
(2)

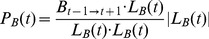
(3)


The length of the projection was then defined for *P_A_*(*t*) and *P_B_*(t) as *SV* for players A and B (namely, *SV_A_* and *SV_B_*, respectively). These values represented each player’s velocity while stepping toward (<0) or away from (0>) the opponent.

#### Relative phase

To calculate the lag between both players’ phases (ΔΦ*_AB_*(*t*)), we deconstructed each player’s *SV* time series into real and imaginary parts using the Hilbert transform, formulated as equations (4) and (5):
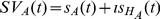
(4)

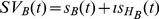
(5)


Finally, ΔΦ*_AB_* (*t*) was obtained by equation (6) [36], [37]:
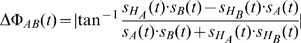
(6)


The relative phase was divided into nine ranges from 0° to 180° (phase regions: 0–20°, more than 20–40°,... more than 160–180°) to maintain consistency with previous studies (e.g. [12], [14]).

#### Possible striking distance

Possible striking distance was calculated using equation (1).

### Statistical Processing

Only the data on interpersonal distances that fell between 0.06 and 3.70 m were statistically tested to preserve a given amount of data for each match. Percentages were calculated based on the interpersonal distance and the relative phase in a match and were then subjected to an angular transformation. These data were tested using repeated-measures ANOVA with the Greenhouse–Geisser correction. The Bonferroni method was used in multiple comparisons.

## Results

### Preferred Interpersonal Distance

**Figure 2:**
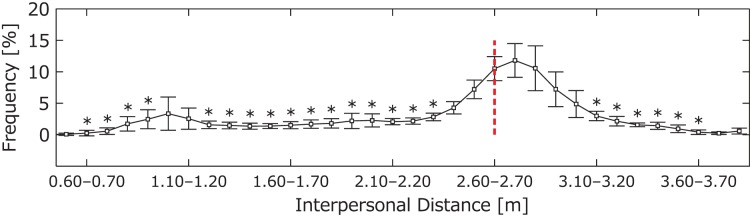
Frequency of interpersonal distance. The frequency of interpersonal distance per 0.10 m in each match was calculated, and the average and standard deviation are shown (0.50–3.90 m). For example, an interpersonal distance in the 0.60–0.70-m range means that the data fell between 0.60 and 0.70 m. The asterisks (*p<.05) above the line indicate distances that were significantly less frequent than were those within the range of 2.50–2.80 m. The red dotted line indicates the average of possible striking distances.

Figure 2 shows the average frequency of interpersonal distance in 12 matches. We observed two peaks, at 1.00–1.10 m and at 2.70–2.80 m. The one-way repeated-measures analysis of variance (ANOVA) of distance regions revealed a significant main effect (*F*(2.84, 31.23) = 58.03, *p*<0.01), and the results of the multiple-comparisons analysis are shown in Figure 2.

The data frequencies around these peaks were 11.64% at 0.80–1.30 m and 56.45% at 2.40–3.10 m, which accounted for 68.09% of the total. The data clearly demonstrate that the players tended to stay within their preferred interpersonal distances and executed their offensive and defensive movements during these matches based on these distances.

### Relative Phase of Players’ Step toward–away Velocity

**Figure 3:**
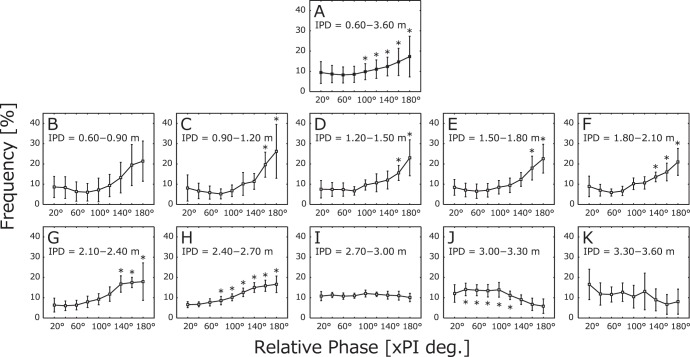
Frequency of relative phase at each interpersonal distance. The frequency of relative phase per 0.30 m was calculated for each match, and the averages and standard deviations are indicated. **A**. Total frequency at all distances (0.60–3.60-m). **B–K**. Partial frequencies at each distance (0.60–0.90 m, 0.90–1.20 m,... 3.30–3.60 m). Relative phases were divided into nine ranges (20°: 0–20°, 40°: 20–40°,... 180°: 160–180°). The black asterisks (*p<.05) above the line indicate distances that were significantly more frequent than were those at 20° and/or 40°, and the asterisks below the line indicate distances that were significantly more frequent than those at 160° and/or 180°.

The average frequency of the relative phase during the 12 matches is indicated in Figure 3A. We observed a slight tendency for players’ movements to be synchronized in the anti-phase as a whole. A one-way repeated-measures ANOVA of phase ranges revealed a significant main effect (*F*(1.60, 17.57) = 23.35, *p*<.01). The results of multiple comparisons are given in Figure 3A.

Relative phase was altered by a change in interpersonal distance (Fig. 3B–K). A two-way repeated-measures ANOVA of interpersonal distance × relative phase regions revealed a significant interaction (*F*(6.86, 75.43) = 8.31, *p*<.01). The results of the simple main effect tests are given in Figure 3B–K. These results indicate that the relative phase showed clear trends toward anti-phase synchronization at near distances, i.e., those closer than 2.70 m, and toward in-phase synchronization at far distances, i.e., those farther than 3.00 m. This in- and anti-phase transition had a regular pattern in that distance provoked the phase transition and corresponded to the preferred interpersonal distance at around 2.70–2.80 m.

**Figure 4:**
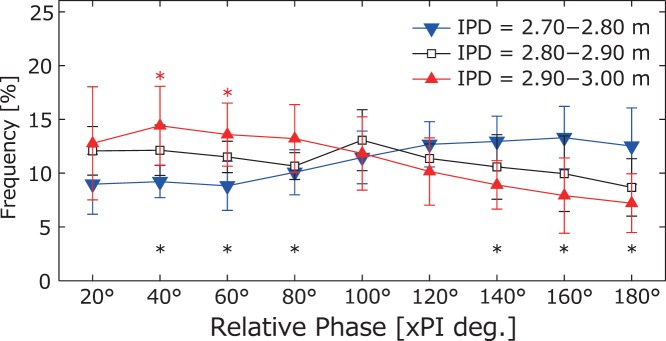
Frequency of the relative phase at interpersonal distances of 2.70–3.00 m. The frequencies of the relative phase per 0.10-m interval at interpersonal distances of 2.70–3.00 m were calculated, and the averages and standard deviations are presented (2.70–2.80 m, 2.80–2.90 m, 2.90–3.00 m). Relative phases were divided into nine ranges (20°: 0–20°, 40°: 20–40°,... 180°: 160–180°). The red asterisks above the red line indicate distances that were significantly more frequent than were those at 160° and/or 180°. The black asterisks below the lines indicate significant differences between 2.70–2.80 m and 2.90–3.00 m in each relative-phase range.

We focused on the distance range between 2.70 and 3.00 m to fully analyze the phase transition. The frequency of the relative phase in the distance per 0.10-m ranges is indicated in Figure 4. A two-way repeated-measures ANOVA of interpersonal distance × relative phase regions revealed a significant interaction (*F*(4.85, 52.97) = 9.05, *p*<.01). The results of the simple main effect tests are given in Figure 4. Surprisingly, the relative phase at this important distance transformed anti-phase synchronization to in-phase synchronization based on a minimal 0.10-m difference in distance. These results clearly show that a higher anti-phase synchronization occurred at 2.70–2.80 m than at other distances, and this was switched to in-phase synchronization at 2.90–3.00 m, setting a boundary at 2.80–2.90 m. Thus, 2.80–2.90 m was the critical interpersonal distance and was the distance needed to change the kendo players’ movements on a slight 0.10-m scale.

### Possible Striking Distance

The players’ possible striking distance averaged 2.65 m, which is indicated by the red dotted line in Figure 2. The standard deviation of 0.06 m was only 2.3% away from the interpersonal distance. These data suggest that all players had an ability to strike any opponent equally from around this distance.

## Discussion

### Preferred Interpersonal Distance

The results regarding the interpersonal distance representing the relationship between two players’ positions revealed preferred interpersonal distances in kendo matches, which were represented as clear peaks at 1.00–1.10 and at 2.70–2.80 m.

The interpersonal distance around 1.00–1.10 m represented the situation in which the distances between two opponents were at their closest and where they continued contacting each other (called tsubazeriai), similar to clinches in boxing matches. The player with a long shinai had to move backward to strike the opponent in these situations. Our data showed 1.00 (*SD* of 1.29) instances of striking and 0.00 points per a match. In fact, the players executed few striking movements in this context, and they primarily separated and returned at around 2.70–2.80 m. Thus, it may be that executing offensive techniques and scoring a point by stepping away are less likely at around 1.00–1.10 m than at around 2.70–2.80 m.

The interpersonal distance between two players of about 2.70–2.80 m represented a neutral posture that allowed a balance between offensive and defensive techniques with maintenance of a ready stance (kamae). To efficiently strike the opponent, the player needed to move forward from approximately 2.70–2.80 m. On the defensive end, the player trying to prevent his opponent from striking needed to maintain a safety space to maintain the interpersonal distance. In general, the smaller the interpersonal distance, the less time needed for the striking movement. With this rule in mind, the task difficulty for the offensive players was gradually reduced as they approached from a far distance. Viewed from a different angle, as the interpersonal distance gradually shortened, the task difficulty for the defensive players increased gradually owing to the decline in the available reaction and movement times. In this situation, the gain and loss of offensive and defensive movements resulted in a disturbance of the normal balance. In general, the players were able to continuously maintain the interpersonal distance to balance gain and loss.

It is possible that a striking distance of 2.65 m was consistent with the preferred interpersonal distance. However, the 0.05–0.15-m difference between the peak value of the preferred distance and the possible striking distance indicates that players tended to engage in subtle maneuvers while maintaining a safety margin for defensive movements [38], [39]. This phenomenon corresponds to the regular variations in the movements of several animals [40], [41] and humans [1], [38], [42], [43] and in sports settings [39], [44] caused by changes in task constraints and visual information. Thus, an interpersonal distance of around 2.70–2.80 m was optimal for subtle maneuvers in preparation for the next striking or defensive movement. The distance, therefore, constitutes the preferred interpersonal distance necessary to maintain a balance between the gain and loss of offensive and defensive movements. Thus, the distance between 1.10 and 2.70 m may be the distance at which players are forced to quickly return to the distances that they prefer and that are appropriate for the task.

### Relative Phase as an Order Parameter and Interpersonal Distance as a Control Parameter

Surprisingly, the relative-phase data, which represent the relationships or coordination between two players’ movements, indicated that the players switched openly and regularly between the in- and anti-phase synchronizations as an order parameter by changing the interpersonal distance as the control parameter. Thus, the phase-transition phenomenon demonstrated in other studies investigating behavioral dynamics in cooperative tasks is also present in kendo matches. The following section compares three sample interpersonal distances (closer than 2.80 m, 2.80–2.90 m, farther than 2.90 m; see Fig. 4) with the relative phase:

1) At distances closer than 2.80 m, including the possible striking distance of 2.65 m, one player would try to execute a strike attempt and step forward, while the other player would try to avoid the strike by stepping backward. This resulted in the players coordinating their interpersonal distances in an anti-phase synchronization. 2) At distances farther than 2.90 m, the two players could not strike each other and, as a result, defensive movements were not necessary. Consequently, this task was not organized, and the players would then approach each other with in-phase synchronization. 3) At distances of 2.80–2.90 m, i.e., 0.15–0.25 m from possible striking distance, interpersonal distance was critical. At these distances, players had to coordinate their movements using the movement strategies discussed above for distances closer than 2.80 m and farther than 2.90 m. This indicates that the players would carry out a variety of movements in order to make subtle offensive and defensive maneuvers actively and passively at those distances. These maneuvers appear to form the clearest peak of preferred interpersonal distance, around 2.70–2.80 m.

Consistent with previous observations of intra- and interpersonal coordination modes [2]–[4], [8] (see Fig. S1A), these changing task constraints resulted in a phase transition of coordination modes (in- and anti-phase) between the two players. It should be noted that the phase transition was not the result of gradual or linear changes in the control parameter, as is the case in cooperative tasks. Rather, phase transitions switched continuously according to abrupt changes in interpersonal distance [45]–[48] (see Fig. S1B). The emergence of this rapid switching dynamic could be a result of expert players’ ability to change their movements in a very short time. These players would understand the need for minute changes on a 0.10-m scale and, consequently, switch their movements regularly to appropriate directions or synchronizations.

It may be confusing that all distances discussed in regard to phase transition contain both in- and anti-phase synchronizations. Previous studies of postural coordination have reported that the relative phase between the ankle and hip motion toward the sagittal plane switched between in- and anti-phase coordination when the moving frequency of the target changed [49], [50]. In a similar manner, the kendo players’ movements switched between the two coordination modes at a critical interpersonal distance. These results have also been reported in studies of competitive tasks [26], [27], [30]–[32]. This phenomenon may be explained by the fact that decreases in the interpersonal distance lead players to switch from in- to anti-phase coordination and *vice versa*, forming a pattern that meets the seemingly opposing goals of executing offensive and defensive maneuvers simultaneously. However, it remains unclear whether this phenomenon was caused by a switching manifold [45]–[48] or by a coupled harmonic oscillator [34].

### General Discussion

We explored the behavioral dynamics of the interpersonal opponent task by examining kendo matches played by expert players. The results indicated that players exhibited two preferred interpersonal distances. The most preferred distance enabled them both to be ready to execute and to actually execute both striking and defensive movements; this distance corresponded to the sum of the possible striking distances plus the safety margin. The change in interpersonal distance was the control parameter and clearly represented the switch in players’ stepping toward/away from the anti-phase synchronization at distances less than 2.80 m and in players’ stepping toward/away from the in-phase synchronization at distances greater than 2.90 m. In terms of accuracy, abrupt phase switching was instantaneously and continuously executed at a 0.10-m scale. This phase-transition phenomenon was able to describe the essence of a whole kendo match in that it described subtle changes in interpersonal distance. It might be that the behavioral dynamics underlying the process of the two expert players were linked by visual information. Indeed, visual feedback is common in other interpersonal competitive tasks, especially those involving opponent tasks.

Previous studies evaluating behavioral dynamics of competitive tasks have observed only parts of sport games or situations (e.g., [51]–[53]). To our knowledge, few studies have elucidated the ‘hidden’ dynamics in natural, complex, and continuous sporting behaviors. Thus, the current data provide new evidence that clear and hidden dynamics are found in a sport setting when it is examined in its entirety. Surprisingly, the synchronization mode switched at interpersonal distances of only 0.10 m in kendo.

These findings have profound implications not only for other sport activities but also for a variety of human social interactions. Switching between in-phase and anti-phase modes may be common in interpersonal competitive situations [54]. Additionally, the regulation of the interpersonal distances separating three people during sports activities is a critical factor for synchronization [55]. Furthermore, the four-way classification of distance zones in social interactions (intimate, personal, social, and public) suggests that humans change their behavior according to the interpersonal distance that separates them from others [1]. Indeed, only slight differences in interpersonal distance would be expected to yield completely different behavioral patterns in any social situation.

## Supporting Information

Figure S1 **Mean, SD, and duration of each relative phase in each interpersonal distance.** A. Blue squares indicate the mean relative phase, and the inclination depicts the characteristics of abrupt phase switching at around 2.70–2.90 m. Red squares show the *SD*s of each relative phase at each interpersonal distance (0.60–0.90 m, 0.90–1.20 m,... 3.30–3.60 m). B. Cumulative histogram of the duration of each relative phase (20°: 0–20°, 40°: 20–40°,... 180°: 160–180°) for 2.70–2.80 m (blue triangles), 2.80–2.90 m (green squares), and 2.90–3.00 m (red triangles), which are critical interpersonal distances for switching coordination modes. Durations of 0.1–0.5 s accounted for a considerable proportion of all the data, which indicates that the two players rarely coordinated using a relative phase mode more than 0.5 s.
(EPS)

Text S1 **A supplementary measure of strike-movement time.** Players’ striking movements in matches were measured to identify players’ physical constraints and the task properties of kendo. The strike movements were measured during the tobikomi-men (lunge-and-strike) movement. Players frequently attempted to strike an opponent’s head (men) from an interpersonal distance of 2 m or more. Movement time was recorded from the start of swinging the shinai upward or the start of moving the right foot forward to the end of swinging of the shinai downward. This strike movement was selected because it required long movement times and was frequently executed in matches. However, even using the long strike movement, the overall movement time of 72 total strikes was very short.
(DOC)
