# Supplementary material for: A Critical Interpersonal Distance Switches between Two Coordination Modes in Kendo Matches
Source: PLoS One. 2012 Dec 20;7(12):e51877. doi: 10.1371/journal.pone.0051877 (PMC3527480; doi:10.1371/journal.pone.0051877)
Supplement: Text S1 — A supplementary measure of strike-movement time. Players’ striking movements in matches were measured to identify players’ physical constraints and the task properties of kendo. The strike movements were measured during the tobikomi-men (lunge-and-strike) movement. Players frequently attempted to strike an opponent’s head (men) from an interpersonal distance of 2 m or more. Movement time was recorded from the start of swinging the shinai upward or the start of moving the right foot forward to the end of swinging of the shinai downward. This strike movement was selected because it required long movement times and was frequently executed in matches. However, even using the long strike movement, the overall movement time of 72 total strikes was very short. (DOC) [file pone.0051877.s002.doc]

Text S1. A supplementary measure of strike-movement time. Players’ striking movements in matches were measured to identify players’ physical constraints and the task properties of kendo. The strike movements were measured during the tobikomi-men (lunge-and-strike) movement. Players frequently attempted to strike an opponent’s head (men) from an interpersonal distance of 2 m or more. Movement time was recorded from the start of swinging the shinai upward or the start of moving the right foot forward to the end of swinging of the shinai downward. This strike movement was selected because it required long movement times and was frequently executed in matches. However, even using the long strike movement, the overall movement time of 72 total strikes was very short.
